# A phase II randomized clinical trial on cerebral near-infrared spectroscopy plus a treatment guideline versus treatment as usual for extremely preterm infants during the first three days of life (SafeBoosC): study protocol for a randomized controlled trial

**DOI:** 10.1186/1745-6215-14-120

**Published:** 2013-05-01

**Authors:** Simon Hyttel-Sorensen, Topun Austin, Frank van Bel, Manon Benders, Olivier Claris, Eugene Dempsey, Monica Fumagalli, Gorm Greisen, Berit Grevstad, Cornelia Hagmann, Lena Hellström-Westas, Petra Lemmers, Jane Lindschou, Gunnar Naulaers, Wim van Oeveren, Adelina Pellicer, Gerhard Pichler, Claudia Roll, Maria Skoog, Per Winkel, Martin Wolf, Christian Gluud

**Affiliations:** 1 Department of Neonatology, Rigshospitalet, Blegdamsvej 9, Copenhagen, DK-2100, Denmark; 2 Rosie Maternity Hospital Cambridge University Hospitals NHS Foundation Trust Hills Road, Cambridge, CB2 0SW, UK; 3 Wilhelmina Children’s Hospital, Universitair Medisch Centrum Utrecht, KE 04.123.1, PO Box 85090, Utrecht, 3508 AB, The Netherlands; 4 Department of Neonatology, Hopital Femme Mere Enfants, 59, Boulevard Pinel, Bron, 69500, France; 5 Department of Paediatrics and Child Health, University College Cork, College Road, Cork, Ireland; 6 NICU, Fondazione IRCCS Ca’ Granda Ospedale Maggiore Policlinico Milan, Via della Commenda 12, Milan, IT- 20122, Italy; 7 Copenhagen Trial Unit, Centre for Clinical Intervention Research, Rigshospitalet, Blegdamsvej 9, Copenhagen, DK-2100, Denmark; 8 Division of Neonatology, University of Zurich, Zurich, 8091, Switzerland; 9 Department of Women’s and Children’s Health Uppsala Universitet, University Hospital, Uppsala, 751 85, Sweden; 10 Department of Neonatology, University Hospital, Uppsala, 751 85, Sweden; 11 Katholieke Universiteit Leuven, Herestraat 49, Leuven, 3000, Belgium; 12 Haemoscan B.V, Stavangerweg 23, Groningen, JC, 9723, The Netherlands; 13 Department of Neonatology, La Paz University Hospital, Paseo de la Castellana 261, Madrid, 28046, Spain; 14 Department of Pediatrics, Medical University of Graz, Auenbruggerplatz 30, Graz, Austria; 15 Department of Neonatology and Paediatric Intensive Care, Vest Children’s Hospital Datteln, University Witten-Herdecke, Dr.-Friedrich-Steiner-Str. 5, Datteln, 45711, Germany; 16 Biomedical Optics Research Laboratory, Division of Neonatology, University Hospital Zurich, Frauenklinikstr 10, Zurich, 8091, Switzerland

**Keywords:** Randomized clinical trial, Preterm, Near infrared spectroscopy, Protocol

## Abstract

**Background:**

Every year in Europe about 25,000 infants are born extremely preterm. These infants have a 20% mortality rate, and 25% of survivors have severe long-term cerebral impairment. Preventative measures are key to reduce mortality and morbidity in an extremely preterm population. The primary objective of the SafeBoosC phase II trial is to examine if it is possible to stabilize the cerebral oxygenation of extremely preterm infants during the first 72 hours of life through the application of cerebral near-infrared spectroscopy (NIRS) oximetry and implementation of an clinical treatment guideline based on intervention thresholds of cerebral regional tissue saturation rStO_2_.

**Methods/Design:**

SafeBoosC is a randomized, blinded, multinational, phase II clinical trial. The inclusion criteria are: neonates born more than 12 weeks preterm; decision to conduct full life support; parental informed consent; and possibility to place the cerebral NIRS oximeter within 3 hours after birth. The infants will be randomized into one of two groups. Both groups will have a cerebral oximeter monitoring device placed within three hours of birth. In the *experimental group*, the cerebral oxygenation reading will supplement the standard treatment using a predefined treatment guideline. In the *control group*, the cerebral oxygenation reading will not be visible and the infant will be treated according to the local standards. The primary outcome is the multiplication of the duration and magnitude of rStO_2_ values outside the target ranges of 55% to 85%, that is, the ‘burden of hypoxia and hyperoxia’ expressed in ‘%hours’. To detect a 50% difference between the experimental and control group in %hours, 166 infants in total must be randomized. Secondary outcomes are mortality at term date, cerebral ultrasound score, and interburst intervals on an amplitude-integrated electroencephalogram at 64 hours of life and explorative outcomes include neurodevelopmental outcome at 2 years corrected age, magnetic resonance imaging at term, blood biomarkers at 6 and 64 hours after birth, and adverse events.

**Discussion:**

Cerebral oximetry guided interventions have the potential to improve neurodevelopmental outcome in extremely preterm infants. It is a logical first step to test if it is possible to reduce the burden of hypoxia and hyperoxia.

**Trial registration:**

ClinicalTrial.gov, NCT01590316

## Background

Every year in Europe about 25,000 infants are born extremely preterm. They are at high risk of death and those surviving may develop cerebral impairment; mortality is about 20% [1], and about 25% of survivors live with either cerebral palsy or low intelligence quotient [2,3].

All organs are immature when an infant is born more than 12 weeks before term. The immaturity and functional limitations of the brain, lungs, heart, intestine, kidneys, liver, and endocrine system contribute to acute condition of the extremely preterm infant. For instance, the immaturity of the brain can result in death or neuropsychological deficits such as cerebral palsy and cognitive deficits [4]. The most easily identifiable type of brain damage in extremely preterm infants is periventricular-intraventricular brain hemorrhage (PV-IVH) [5]. The severity varies; however, in its most severe form it predicts high probability of cerebral palsy, hydrocephalus, or death [6,7]. Periventricular leucomalacia (PVL) is non-hemorrhagic white matter damage and a strong predictor of cerebral palsy [8].

The mechanisms of brain damage in preterm infants are complex. Some of the mechanisms are evoked before birth or even before the start of delivery such as a fetal inflammatory response induced by infection ascending to the fetal membranes [1]. Also, late effects such as insufficient nutrition and poor growth during the first months of life may play a role [9]. The first days after birth are likely to be of particular importance, and the circulatory adaption to birth is problematic. Moreover, birth is a change from a state of low oxygen pressure (‘Mount Everest *in utero*’) to a state of ‘normoxemia’. The following postnatal factors have been shown or are thought to be associated with brain injury: respiratory distress syndrome [10], hypocapnia due to inadvertent hyperventilation [11], low blood pressure [12], perturbations in arterial and venous pressure [13], and low cerebral blood flow [14]. In addition, clinical and experimental evidence suggest that hyperoxygenation is dangerous due to lack of a developed antioxidant defense system [15].

Preventative measures are key to reduce mortality and morbidity in an extremely preterm population. Near-infrared spectroscopy (NIRS) has been used to monitor tissue oxygenation since the mid-1980s. The quantification of the tissue oxygen saturation of hemoglobin (rStO_2_) in percentage from 0 to 100% has been possible for 10 years. Data from almost 400 preterm infants using NIRS oximeter INVOS 4100/5100 with the adult SomaSensor (SAFB-SM) showed that the normal ranges of rStO_2_ to be from 55% to 85% (unpublished data from the group of Lemmers and van Bel, Utrecht). Important clinical information can be gained from cerebral NIRS oximetry in neonates. rStO_2_ has been shown to be a predictor of outcome in hypoxic-ischemic encephalopathy [15] and to be low in infants with a patent ductus arteriosus [16]. However, no solid evidence of the clinical utility of NIRS in preterm infants exists. There is a lack of randomized clinical trials. Thus, further research on the benefits and harms of cerebral monitoring using NIRS as a part of clinical management of premature infants is needed.

### Objectives

The primary objective of the SafeBoosC phase II trial is to examine if it is possible to stabilize the cerebral oxygenation of extremely preterm infants during the first 72 hours of life through the application of cerebral NIRS oximetry and implementation of an rStO_2_-specific clinical treatment guideline. We hypothesize that using the treatment guideline to respond to cerebral monitoring readings outside the target range will reduce the burden of hypoxia and hyperoxia and consequently reduce brain injury of extremely preterm newborns.

## Methods/Design

The study will be conducted according to Good Clinical Practice guidelines and the Declaration of Helsinki. The protocol will be approved by the Ethics Committees of each participating center involved and if necessary by national law by the relevant competent authority. Written informed consent must be obtained before inclusion of an infant.

### Ethical approvals

By 01-March-2013 the study had been approved by Clinical Research Ethics Committee of the Cork Teaching Hospitals (EMC 3 (xxx) 03/07/12); NRES Committee East of England - Cambridge South (12/EE/0329); Ethikkommission Medizinische Universität Graz (24-261 ex (11/12)); Commissie Medische Ethiek Van De Universitaire Ziekenhuizen Kuleuven (ML8536); De Videnskabetiske Komiteer - Region Hovedstaden (H-4-2012-028); CPP Sud-Est III - Lyon (HCL/P 2012.728); Direzione Scientifica - Comitato di Etica - The IRCCS Fondazione Ca’ Granda Ospedale Maggiore Policlinico (549/12 - all.3); Medisch Ethische Toetsingscommissie - Universitair Medisch Centrum Utrecht) (WAG/rc/12/036774), and Comité Ético de Investigación Clínica del Hospital Universitario ‘La Paz’ de Madrid (15/2011).

### Burden of hypoxia and hyperoxia

The burden of hypoxia is the multiplication of duration and magnitude of rStO_2_ values below 55%. The burden of hyperoxia is the multiplication of duration and magnitude of rStO_2_ values above 85%. The sum of the burden of hypoxia and hyperoxia is expressed in ‘%hours’.

### Trial design

The SafeBoosC is an investigator-initiated randomized, blinded, multinational, phase II feasibility clinical trial designed to include extremely preterm infants from 12 European countries.

### Population

The trial population is extremely premature infants.

### Inclusion criteria

The inclusion criteria are: neonates born more than 12 weeks preterm (gestational age up to 27 weeks and 6 days), decision to conduct full life support, parental informed consent, and possibility to place the cerebral NIRS oximeter within 3 hours after birth.

### Exclusion criteria

Exclusion criteria are a decision not to provide life support or lack of informed consent.

### Randomization

Web-based randomization is conducted centrally at the Copenhagen Trial Unit according to a computer-generated allocation sequence with a varying block size concealed for the investigators. The allocation is stratified for gestational age (<26 weeks or ≥26 weeks). Singleton infants will be randomized individually. Twins will be randomized as a ‘pair’; that is, both siblings will be allocated to the same treatment group. If not all infants from a multiple birth can be included due to lack of equipment, it is allowable to include as many as possible always starting with the infant born last and then the infant born second last and so forth.

### Intervention

The preterm infants will be randomized into one of two groups; both groups will have a cerebral oximeter monitoring device placed within three hours of birth. In the *experimental group*, the cerebral oxygenation reading will be visible and the infant will be treated accordingly using a predefined treatment guideline For the *control group*, the cerebral oxygenation reading will NOT be visible and the infant will be treated according to local guidelines.

### Devices for cerebral NIRS monitoring

Cerebral oximetry by NIRS in extremely preterm infants has no reference standard, however, this clinical investigation will use the INVOS 5100c™ with the adult SomaSensor™ as reference for NIRS device eligibility. The application of the adult sensor in this population is considered safe as no serious adverse effects relating to the oximeter, such as skin burns *etcetera*, were encountered in these infants. Device eligibility is tested by comparison of absolute values, reproducibility and sensitivity to changes in oxygenation on the adult arm [17]. Absolute values and dynamic range within 5 percentage points of the INVOS and reproducibility better than 6% are the predefined thresholds. So far eligible devices for the present trial include INVOS 5100C™ with Adult SomaSensor™, NIRO 200NX™ with small probe-holder, and NONIN EQUANOX 7600™ with adult sensor Model 8004CA™ [17]. The use of adult sensors in the neonates will need approval in some countries from a competent authority.

The devices will be locked in a box and all data will be sent to a laptop real time. Software has been developed that shows the current rStO_2_ with trend lines and saves the data as comma separated values. Accordingly, rStO_2_ data in both the experimental and the control group will be collected; however, rStO_2_ in the control group will not be visible to the health-care professionals. Moreover, all trial-related interventions and sensor re-positioning, as well as physiological parameters such as blood pressure, mean airway pressure, *etcetera,* will be registered by the health-care professionals.

### Treatment guideline

The treatment guideline aims at controlling the oxygen delivery to the brain. In brief, it suggests a non-prioritized number of possible interventions if the rStO_2_ is out of range. For ventilatory support, the interventions could be to change the inspiratory oxygen fraction, the mean airway pressure, or the minute ventilation. For hemodynamic support, the possible interventions include inotropics/vasopressors to increase blood pressure, medical closure of the ductus arteriosus, or red blood cell transfusion to increase oxygen capacity and intravascular volume. The rationale for and content of the treatment guideline are described in detail in a complementary paper (in press).

### Trial duration

Monitoring by cerebral NIRS oximetry will start as soon as possible or within 3 hours after birth. The intervention will last for 72 hours. The infants will be followed up for 24 months after term date.

### Demographics

Known pregnancy-related risk factors will be registered, for example, preeclampsia, chorioamnionitis, maternal smoking, corticosteroid administration before birth, and multiple births. Moreover, known risk factors during the first days of life, such as gestational age, birth weight, Apgar scores, umbilical cord pH, respiratory distress, mode of ventilatory support, and presence of a patent ductus arteriosus, will also be registered.

### Outcome measures

The primary outcome is the burden of hypoxia and hyperoxia in % hours during the first 72 hours after birth. The secondary outcomes are all-cause mortality at term date, cerebral ultrasound score [18], and interburst intervals on an amplitude-integrated electroencephalogram (aEEG) at 64 hours of life. Exploratory outcomes include blood biomarkers brain fatty acid binding protein (BFABP), neuroketal, and S100β [19-21] at 6 and 64 hours; and serious and non-serious adverse reactions (SARs); burden of hypoxia; burden of hyperoxia; neonatal morbidities including bronchopulmonary dysplasia (BPD), necrotizing enterocolitis (NEC), and retinopathy of prematurity (ROP); brain injury on magnetic resonance imaging (MRI), that is, brain injury score according to Woodward score plus cerebellum scoring [22], volumetric measurements, cortical folding with formation of the sulci, and diffusion tensor imaging; and psychomotor impairment according to neurodevelopmental scales at 24 months after term date (Table 1). Analysis of outcome measures will be blinded to group allocation. When possible the analysis will be centralized.

**Table 1:** Outcome measures of the SafeBoosC phase II trial

**Tools**	**Outcomes**	**Time points**
Primary outcome		
Cerebral NIRS oximeter	• Burden of hypoxia and hyperoxia	• During the intervention until 72 hours after birth
Secondary outcomes		
aEEG/EEG	• Interburst interval (IBI)	• 64 hours after birth
cUS	• Worst Brain injury score (1 to 3) of five cUSs	• At 1 to 4 days after birth
		• At 7 days after birth
		• At 14 days after birth
		• At 35 days after birth
		• At term date
Medical records	• All-cause mortality	Term date
Exploratory outcomes		
Medical records	• Serious adverse reactions	• During the first 7 days of life
	• Non-serious adverse reactions	
Cerebral NIRS oximeter	• Burden of hypoxia	• During the intervention until 72 hours after birth
	• Burden of hyperoxia	
Blood samples	• BFABP	• 6 hours after birth
	• Neuroketal	• 64 hours birth
	• S100β	
Medical records	• Neonatal morbidities:	• Term date
	• NEC stage 2 to 3	
	• ROP stage 3+ and above	
Medical records	Neonatal morbidities:	• At 36 weeks
	• BPD	
aEEG/EEG	• Power in delta band	• 64 hours after birth
	• Power in theta band	
	• Power in alpha band	
	• Power in beta band	
MRI	• Brain injury score (Woodward)	• At term date
	• Volumetric	
	• Cortical folding diffusion tensor imaging	
BSID-III	• Cognitive score	• 24 months after term date
	• Verbal score	
	• Motor score	
ASQ-III	• Total score	• 24 months after term date

aEEG, amplitude-integrated electroencephalogram; ASQ-III, Ages and Stages Questionnaires, Third Edition™; BFABP, brain fatty acid binding protein; BPD, bronchopulmonary dysplasia; BSID-III, Bayley Scales of Infant and Toddler Development, Third Edition™; cUS, cerebral ultrasound; EEG, electroencephalogram; MRI, magnetic resonance imaging; NEC, necrotizing enterocolitis; NIRS, near-infrared spectroscopy; ROP, retinopathy of prematurity.

### Statistical considerations

#### Sample size

In a sample of 23 infants born before 28 weeks of gestation the mean burden of hypoxia and hyperoxia was 76.0%hours ± standard deviation (SD) of 83.2%hours. With a 50% reduction of the area outside the normal range of oxygenation in %hours in the experimental group compared with the control group as the minimal clinically significant difference, a SD of the area outside the normal range of 83.2%hours, a type I error (alpha) of 5%, and a type II error of 0.05 (power of 95%) the inclusion of 75 extremely preterm infants in the experimental group and 75 extremely preterm infants in the control group is required. However the prevalence of twins among extremely preterm infants is about 30%. The within-cluster correlation diminishes the statistical power. To account for this the sample size needs to be multiplied by the ‘design effect’ [23]:

$$\text{Design}\;\text{effect}=1+\left(n’–1\right)\times \text{ICC}$$

ICC = intraclass correlation coefficient

n’ = average cluster size

The twin ICC is unknown for cerebral oximetry. A pragmatic solution is to increase the sample size to 166 in the present study with a continuous outcome and only about 30% of infants in clusters of two. This corresponds to an ICC of 0.33.

### Data analysis

All analyses will be intention to treat analyses conducted blinded with two-sided tests at the 0.05 level of significance. A fully specified statistical analysis plan for the main analysis, including programming details, will be written and approved before unmasking.

The statistical analysis is sophisticated by the fact that up to 30% of the births may be twin births where both siblings will be randomized to the same arm creating multiple clusters of two observations that may be correlated. Shaffer *et al.*[24] found that for this type of data without covariates added a mixed effects model with random intercept is the preferred model for continuous outcome data while ordinary logistic regression is preferred for binary data. Sauzet *et al*. [25] studied the effect of including adjusting covariates in the model for a continuous outcome variable. They found not negligible and similar bias for estimated coefficients whether a mixed model or a linear regression model was used. Standard errors were underestimated using linear models thus inflating the type I error.

A mixed model with random intercept for continuous outcome variables and ordinary logistic regression for binary (and ordinal) outcome variables will be used. The analyses will be adjusted for the protocol specified variables (gestational age category and trial site indicator) and (following multiple imputations if necessary) will be the primary results. Since the intracluster correlation may cause parameter estimate bias in the presence of covariates (other that the intervention indicator) for continuous outcome variables an unadjusted analysis will also be done. If the results of the adjusted and unadjusted analysis differ markedly the results will be discussed. The effect of covariates is unknown for binary outcome variables.

If for a specified statistical model to be used in one of the above analyses of primary and secondary outcome measures Little’s test is significant (*P* <0.05) and the percent missing cases >5%, multiple imputations will be applied to adjust for values missing at random.

To assess the spectrum of potential bias resulting from data missing not at random the following sensitivity analysis will be done for the primary outcome measure: let A be the group with a beneficial effect (low outcome value) as compared to the other group (group B), min be the minimum value, and max the maximum value in the material. Two estimates of the coefficient (c) of the intervention indicator will then be calculated where missing values in A are replaced by the maximum value found in the material and missing values in B are replaced by minimum value found in the material and vice versa. Since the imputation may impact the standard error of the parameter estimate in an unpredictable way the standard error of the primary analysis will be used in each case to test if the estimate deviates significantly from 0.

The null hypothesis corresponding to the primary outcome measure is tested at the 0.05 level of significance. If the test is significant the three secondary outcomes (interburst interval (IBI) at 64 hours, cerebral ultrasound (cUS), all cause mortality at term) will be tested using alpha = 0.05 and Hommel’s procedure as adjustment for multiplicity [26]. The statistical analysis will be done using SPSS™ version 17 or later (IBM Corporation, New York, USA).

### Safety

Serious adverse reactions and suspected unexpected serious adverse reactions (SUSARs) will be recorded and reported according to the ISO14155:2011 ‘Clinical Investigation of Medical Devices’ [27] to the appropriate competent authorities and ethics committees. Since the extremely preterm population is a high-risk population it has been decided that only events with a certain/likely/probable relation to the intervention will be reported whereas all other non-serious and serious adverse events will not be reported.

### Ethical considerations

The research question whether monitoring of cerebral oxygen saturation levels in the first 72 hours after birth and subsequent treatment according to predefined guidelines can prevent brain injury and maybe death in extremely preterm infants can only be answered in that very population. There is a potential benefit to the infants in the experimental group. Currently, there are no randomized clinical trials, thus no conclusive evidence, on the benefit of NIRS monitoring in preterm infants, and there is no evidence of risk of harm from cerebral oximetry in this population. Therefore, a small risk of harm from the sensor or manipulation among both the experimental and the control group cannot be excluded. It is thus considered ethically acceptable that the control group receives ‘treatment as usual’. All interventions proposed in the treatment guideline are commonly used in this patient group.

Due to the clinical nature of the trial population, the extremely preterm infants, stress reactions related to the manipulation during positioning and repositioning of the cerebral NIRS oximeter sensors can occur. It is, however, not considered to give substantially more risk or discomfort compared with no intervention.

To minimize parental concern, it has been decided that multiple births will be randomized together and undergo allocation to the same intervention.

To obtain evidence-based knowledge on the benefit and harms of cerebral monitoring using NIRS as part of clinical management of premature infants, a large-scale randomized clinical trial with a clinically relevant outcome, such as survival without neurodevelopmental deficit is needed. The SafeBoosC phase II trial presented here serves as a feasibility trial for such a phase III trial.

## Discussion

SafeBoosC phase II trial tests the hypothesis that cerebral oxygenation in extremely preterm infants can be stabilized to the extent that the burden of hypoxia and hyperoxygenation can be reduced by 50%. Furthermore, the trial includes numerous secondary and exploratory outcomes that explore its feasibility and usefulness in a multinational trial and hence help with the planning of a phase III trial with a clinically relevant and patient-relevant primary outcome.

The trial design of the SafeBoosC phase II has potential problems. Both the interventional and the control group will be monitored with NIRS and will be exposed to the same risks regarding the positioning of the sensor such a burns and increased manipulation of the infant, thus a higher drop out in the control group is possible. Hopefully a low frequency of adverse events and reactions and full support from clinical staff will minimize this problem. Moreover, the poor reproducibility when changing sensor position [17,28] could introduce a bias as the nurses caring for infants in the intervention group will see the rStO_2_ and when the value is out of range may reposition the sensor to try to obtain a within-range value. A standard operating procedure for repositioning will be available and all repositioning will be registered and analyzed to estimate the magnitude of this problem.

Cerebral oximetry has rather poor precision with the present technology. The devices that are commercially available do not give identical values. Furthermore, only a statistically based target range is available, rather than a target range defined by minimal risk of brain injury. All this, however, does not subtract from the relevance of the present trial. The trial will test the possibility of managing cerebral oxygenation, regardless of its precise value or its precise clinical relevance. In contrast to this, a phase III trial is critically dependent on the precision of cerebral oximetry as well as on an appropriate target range.

Brain hypoxia and hyperoxia are one of the several likely causes of neurodevelopmental deficit in extremely preterm infants. Therefore, it is not likely that the risk of deficits can be reduced by more than 5 percentage points - from 25 to 20%. An effect on mortality is not foreseen. Therefore, a phase III trial with survival without neurodevelopment deficit as the primary outcome will have to include about 4000 infants.

In conclusion, cerebral oximetry has the potential to improve neurodevelopmental outcome in extremely preterm infants. It is a logical first step to test if it is possible to reduce the burden of hypoxia and hyperoxia significantly by the combination of cerebral oximetry with a predefined clinical guideline.

## Trial status

Patient recruitment is ongoing (01-nov-2012) and can be accessed at http://www.safeboosc.eu.

## Abbreviations

aEEG: Amplitude-integrated electroencephalogram; ASQ: Ages and Stages Questionnaires Third Edition™; BFABP: Brain fatty acid binding protein; BPD: Bronchopulmonary dysplasia; BSID-III: Bayley Scales of Infant and Toddler Development Third Edition™; cUS: Cerebral ultrasound; EEG: Electroencephalogram; IBI: Interburst interval; ICC: Intraclass correlation; MRI: Magnetic resonance imaging; NEC: Necrotizing enterocolitis; NIRS: Near-infrared spectroscopy; PV-IVH: Periventricular-intraventricular brain hemorrhage; PVL: Periventricular leucomalacia; ROP: Retinopathy of prematurity; SARs: Serious adverse reactions; SUSARs: Suspected unexpected serious adverse reactions.

## Competing interests

The authors declare that they have no competing interests.

## Authors’ contributions

SHS, GG, and CG contributed to the conception and design of the protocol, drafted the manuscript, and will give final approval of the version to be published. LSS, BG, MS, and, JLH have contributed to the conception and design of the protocol, drafted the main protocol, revised the manuscript critically for important intellectual content, and will give final approval of the version to be published. PW has drafted the statistical analysis, revised the manuscript critically for important intellectual content, and will give final approval of the version to be published. TA, FvB, MB, OC, GD, MF, CH, LHW, PL, GN, WvO, GP, AP, CR, and MW have contributed to the conception and design of the protocol, revised the manuscript critically for important intellectual content, and will give final approval of the version to be published. All authors read and approved the final manuscript.
